# Comparative evaluation of pH and Ca ^+^ ion release from MTA on interaction with platelet-rich fibrin and blood clot: an *in vitro* study

**DOI:** 10.12688/f1000research.130227.2

**Published:** 2023-10-30

**Authors:** Sonia Khatri, Sylvia Mathew, Shruthi Nagaraja, Swaroop Hegde, Soumyadeep Ghosh, Kavimalar Ravichandran

**Affiliations:** 1 Conservative Dentistry and Endodontics, Faculty of Dental Sciences, Ramaiah University of Applied Sciences, Bangalore, Karnataka, 560054, India; 2 Conservative Dentistry and Endodontics, Manipal College of Dental Sciences, Manipal, Manipal Academy of Higher Education, Manipal, Karnataka, 576104, India; 3 Conservative Dentistry and Endodontics, Sri Ramakrishna Dental College and Hospital, Coimbatore, Coimbatore, Tamil Nadu, India

**Keywords:** Regenerative Endodontics, Mineral Trioxide Aggregate, Platelet Rich Fibrin

## Abstract

**Background:** ‘Regenerative endodontics’ using host-derived scaffolds and biomaterials (MTA) is popular in the management of teeth with open apex. Alkaline pH and bioactivity contribute to tissue healing and remineralization. We assessed the influence of PRF and Blood Clot on the pH and Ca ^+^ ion release from MTA.

**Methods:** A total of 15 single-rooted human extracted teeth were sectioned at the level of the cementoenamel junction. Based on the type of scaffolds used, samples were divided into three groups. Group 1 (MTA+ PRF), Group 2 (MTA + Blood Clot), Group 3 (control MTA). The prepared specimens were transferred to a fresh falcon tube containing 10mL of distilled water and the collected solutions were analysed for pH and Ca ^+^ ion release at 3h, seventh day and 14 ^th^ day.

**Results:** It was observed that the mean pH and Ca ^+^ ion release were significantly lower in the experimental groups as compared to the control group. Though there was an increase in the pH recorded in Group 1 and 2 at all time periods, the difference was not significant. Ca ^+^ ion release peaked at Day 7 (Group3 > Group2 > Group1) and reduced significantly on the 14 ^th^ day for all groups.

**Conclusions:** Within the limitations of the study, it can be concluded that PRF and blood clot influence the pH and Ca ^+^ ion release from MTA.

## Introduction

‘Regenerative endodontics’ using biomaterials, stem cells, biomimetic scaffold and bioactive growth factors have contributed immensely to the clinical management of teeth with pulp necrosis and underdeveloped roots. Success in the form of regression of apical lesion, continued root maturation in length and thickness may return the tooth to vitality. ^1^ Pulp revascularization techniques have gathered much consideration due to their feasibility, cost-effectiveness and reasonable success rate.

Current regenerative endodontic procedures utilize various host-derived scaffolds such as intracanal blood clots (BC) or platelet substitutes like platelet-rich fibrin (PRF), ^2^ platelet-rich plasma (PRP), which supply necessary signalling molecules with growth factors for tissue regeneration. The alkaline irrigants (sodium hypochlorite), medicaments (triple antibiotic paste and calcium hydroxide) and biomaterials like mineral trioxide aggregate (MTA) or biodentine with different biological properties can also impact the outcome.

On hydration calcium silicates present in MTA produce calcium silicate hydrate and calcium hydroxide. Some of the hydration products such as calcium hydroxide dissociate into Ca ^+^ and OH ions, increasing the pH, contributing to antibacterial activity, osteogenic differentiation and bone formation. ^3^ It has been observed that acidic environments increase the solubility of materials, inhibiting the setting reaction and the sealing ability. ^4^

Calcium ions released from the MTA have been shown to pass through the cell membrane by depolarization or activation of membrane-bound calcium channels stimulating the expression of bone-associated proteins which may contribute to the repair process. ^5^ It can further activate ATP leading to osteoblast and cementoblast differentiation and hard tissue mineralization. ^6^

The regenerative technique should create an environment that favours disinfection and tissue regeneration. Inducing bleeding or use of PRF for facilitating root closure followed by placing a biomaterial may positively or negatively affect the pH and Ca ^+^ release. Knowing the release kinematics can improve our understanding of the regenerative process and help modify treatment strategies. This study thus explored the influence of PRF and BC on the pH and Ca ^+^ ion release from MTA.

## Methods

The study protocol was approved by the Human Research Ethics Committee of M S Ramaiah University of Applied Sciences, Bangalore, Karnataka ethical protocol number (EC-2018/PG/25). Written informed consent was obtained from a healthy volunteer for obtaining fresh blood.

Fifteen previously extracted, single-rooted human teeth were selected. The teeth were sectioned at the level of the cementoenamel junction (CEJ) using a slow-speed diamond disc. ^7^ Only roots with a single canal were selected. The apical end of the sectioned root was further trimmed to obtain a uniform root length of 10mm for all the specimens. The root canal was then prepared using Peeso reamers Number 1–6 (Kerr, Kerr Corporation, Orange, CA) followed by the use of long straight diamond point to achieve a final canal diameter of 4mm. After mechanical instrumentation, the canals were irrigated with 1.5% sodium hypochlorite (20 mL, 5 min) (PERCAN N, SEPTODONT), 5 mL sterile physiological saline (0.9% NaCl) followed by a final rinse with 20 mL of 17% EDTA liquid (PRO Endo EDTA liquid, SS WHITE) and dried using sterile paper points.

### PRF membrane preparation

A 5 mL blood volume was drawn by venipuncture of the antecubital vein from a healthy volunteer and transferred to a 5mL sterile PRF tube (BD vacutainer serum tubes 5mL) without anticoagulant and centrifuged immediately at 3000 revolutions/min (rpm) for 10 min. The resultant PRF clot between the acellular platelet poor plasma and red blood cells was separated out, squeezed on a piece of sterile gauze to obtain PRF membrane. ^8^

### Sample preparation

Coronal end of the root was sealed with wax (modelling wax, PYRAX). MTA (Angelus Indústria de Produtos Odontológicos S/A, Londrina, Brazil) was mixed (one scoop powder was mixed with 0.33 mL of liquid) and condensed into the prepared canal to obtain a thickness of 4 mm and confirmed by a graduated probe. Fifteen samples thus prepared were divided into three groups.

### Group 1 (PRF+MTA)

PRF membrane cut into fragments was placed into the root canal and gently compacted over the MTA using a hand plugger to achieve a thickness of 5 mm.

### Group II (BC+MTA)

Blood drawn by venipuncture of the antecubital vein of a healthy volunteer was introduced into the root canal and left to form a clot. ^9^

### Group III (MTA control)

MTA was mixed according to the manufacturer’s instructions (one scoop of powder was mixed with 0.33 mL of liquid). Cement was then placed into the prepared canal to obtain a thickness of 4 mm and confirmed by a graduated probe.

### Experimental model preparation for Ca ion release and pH

The prepared specimens were transferred to sterile falcon tubes containing 10 ml of distilled water ^7^ and stored at 37 ^o^C and 100% relative air humidity throughout the testing period. At the end of every experimental period (three hours, seven days and 14 days), the specimens were transferred to a fresh falcon tube containing 10 mL of distilled water and the collected solutions were analysed for pH and Ca ^+^ ion release.

### Analysis of pH

The digital pH meter (HI5221 Hanna Instruments Cal Check, United States) was calibrated to pH 7 with standard buffer solution before use. The refillable calomel electrode was placed into the falcon tube containing 10 mL of solution to record the pH. The electrode was washed with distilled water and wiped dry between readings.

### Analysis of Ca ^+^ ion

To determine the release of Ca ^+^ ions, ICP-OES Inductively Coupled Plasma- Optical Emission Spectrometry (Thermo Fisher ICAP 7400 ICP-OES, Radial N. American, USA). equipment was used, owing to its high sensitivity and stability for inorganic analysis. It provides rapid and multi-element analysis of the solutions.

### Statistical analysis

The pH and Ca ^+^ ion release were presented as mean values. One-way ANOVA followed by *post hoc* Bonferroni test were done to determine the significant differences between groups using IBM SPSS software version 22.0 (IBM Corp., Armonk, NY, USA). The results were considered statistically significant if the *p*-value was less than 0.05.

## Results

### pH analysis

The mean pH values of the individual groups at different time periods are presented in Table 1. The control group (MTA) exhibited a mean value of 10.76 at 3h, 11.54 on the seventh day, and 11.16 on the 14 ^th^ day, which was significantly higher than the other groups (p=0.000). pH readings for Group 1 (MTA +PRF) were 7.56 at 3h, 7.78 at seven days which increased to 9.10 on the 14 ^th^ day. Group 2 (BC) presented pH of 6.68 at 3h which increased to 8.64 on the seventh day and 9.28 on the 14 ^th^ day. There was a significant difference in pH between Groups 1 and 2 at 3h and seven days (p < 0.001) but on the 14 ^th^ day there was no significant difference between the groups (p > 0.973).

**Table 1.** pH among the groups using one-way ANOVA followed *post hoc* Bonferroni test at different experimental periods.

Variable	Group	Mean	SD	p value
pH after 3 hours	MTA+PRF	7.56 ^ab^	0.21	<0.001
	MTA+BC	6.86 ^ac^	0.05	
	MTA Control	10.76 ^bc^	0.09	
pH after 7 days	MTA+PRF	7.78 ^ab^	0.11	<0.001
	MTA+BC	8.64 ^ac^	0.13	
	MTA Control	11.54 ^bc^	0.05	
pH after 14 days	MTA+PRF	9.10 ^a^	0.33	<0.001
	MTA+BC	9.28 ^b^	0.30	
	MTA Control	11.16 ^ab^	0.17	

^a,b,c^ Here same pairs of letters on two different mean values denotes a significant difference between that particular pair.

### Calcium ion release

The mean Ca ^+^ ion release of the individual groups at different time periods is given in Table 2. The mean values of Ca ^+^ ions at 3hin the control group (Group 3) was 1.70, MTA +PRF (Group 1) was 0.77 and MTA+ BC (Group2) was 0.72. Group 3 exhibited significantly higher Ca ^+^ ion release as compared to Group 1 and Group 2 at 3h. On the seventh day, MTA+PRF (Group 1) had a Ca ^+^ ion value of 18.39, MTA+ BC (Group 2) recorded a value of 39.20. The control group (Group 3) recorded a maximum value of 203.08, which was significantly higher than both experimental groups. On the 14 ^th^ day Ca ^+^ion release from all groups reduced significantly. The recorded Ca ^+^ ion release of MTA+PRF (Group 1) was 2.01, MTA+ BC (Group 2) was 2.75 and control group (Group 3) had a value of 4.73. The Ca ^+^ ion release from Group 1 and Group 2 was not significantly different for all the experimental time periods. The control group exhibited the maximum Ca ^+^ ion release for all the time periods as compared to the experimental groups.

**Table 2.** Ca ^+^ ion (mg/L) release among the groups using one-way ANOVA followed *post hoc* Bonferroni test for different experimental durations.

Variable	Group	Mean	SD	p value
Ca ^+^ 3 hours	MTA+PRF	0.77 ^a^	0.13	0.002
	MTA+BC	0.72 ^b^	0.12	
	MTA Control	1.70 ^ab^	0.58	
Ca ^+^ 7 days	MTA+PRF	18.39 ^a^	4.57	<0.001
	MTA+BC	39.20 ^b^	8.78	
	MTA Control	203.08 ^ab^	28.66	
Ca ^+^ 14 days	MTA+PRF	2.01 ^a^	0.71	<0.001
	MTA+BC	2.75 ^b^	0.51	
	MTA Control	4.73 ^ab^	0.41	

^a,b^ Here same pairs of letters on two different mean values denotes a significant difference between that particular pair.

**Figure 1.**
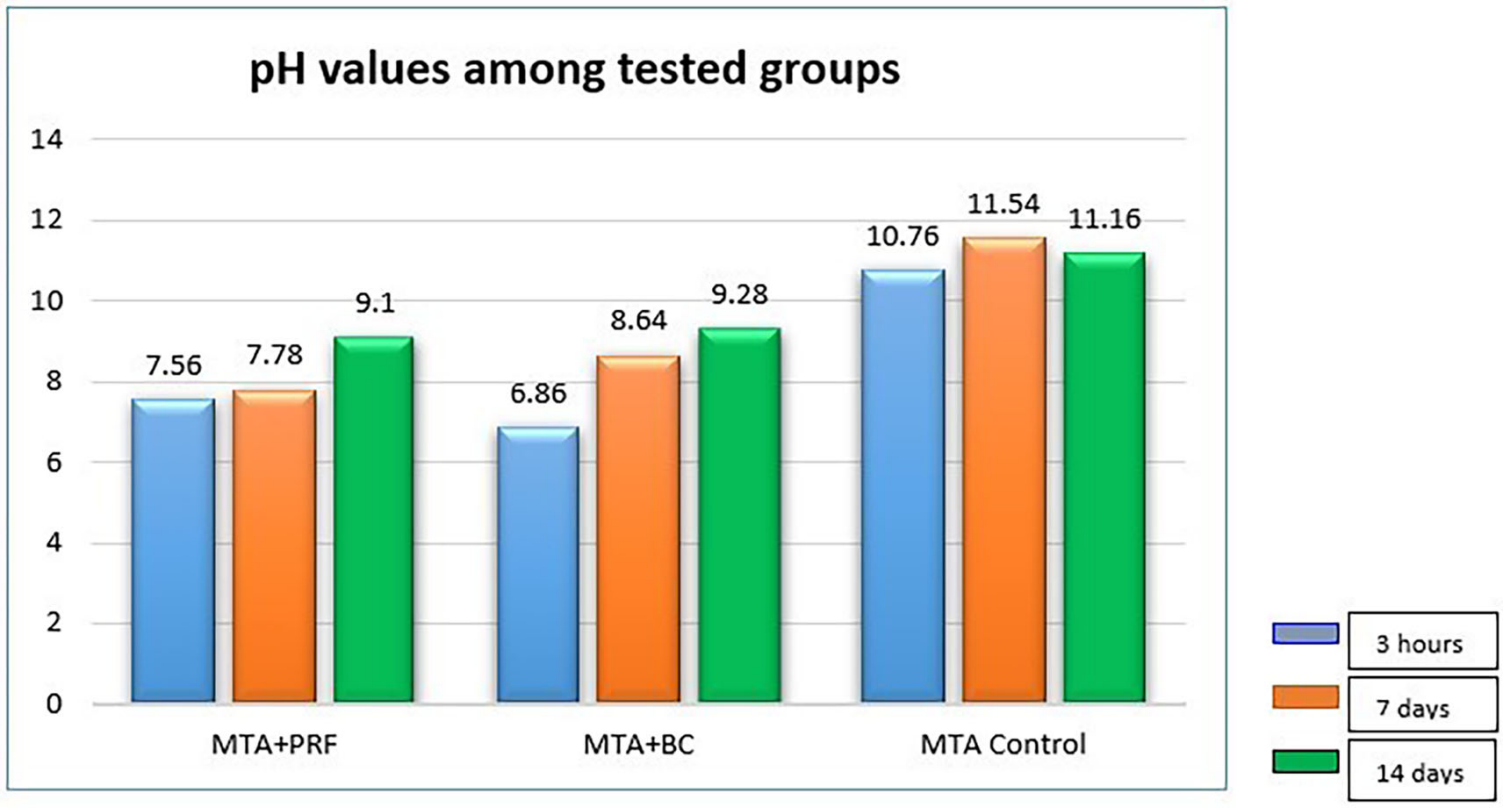
Bar graph representing pH among the groups at different experimental periods.

**Figure 2.**
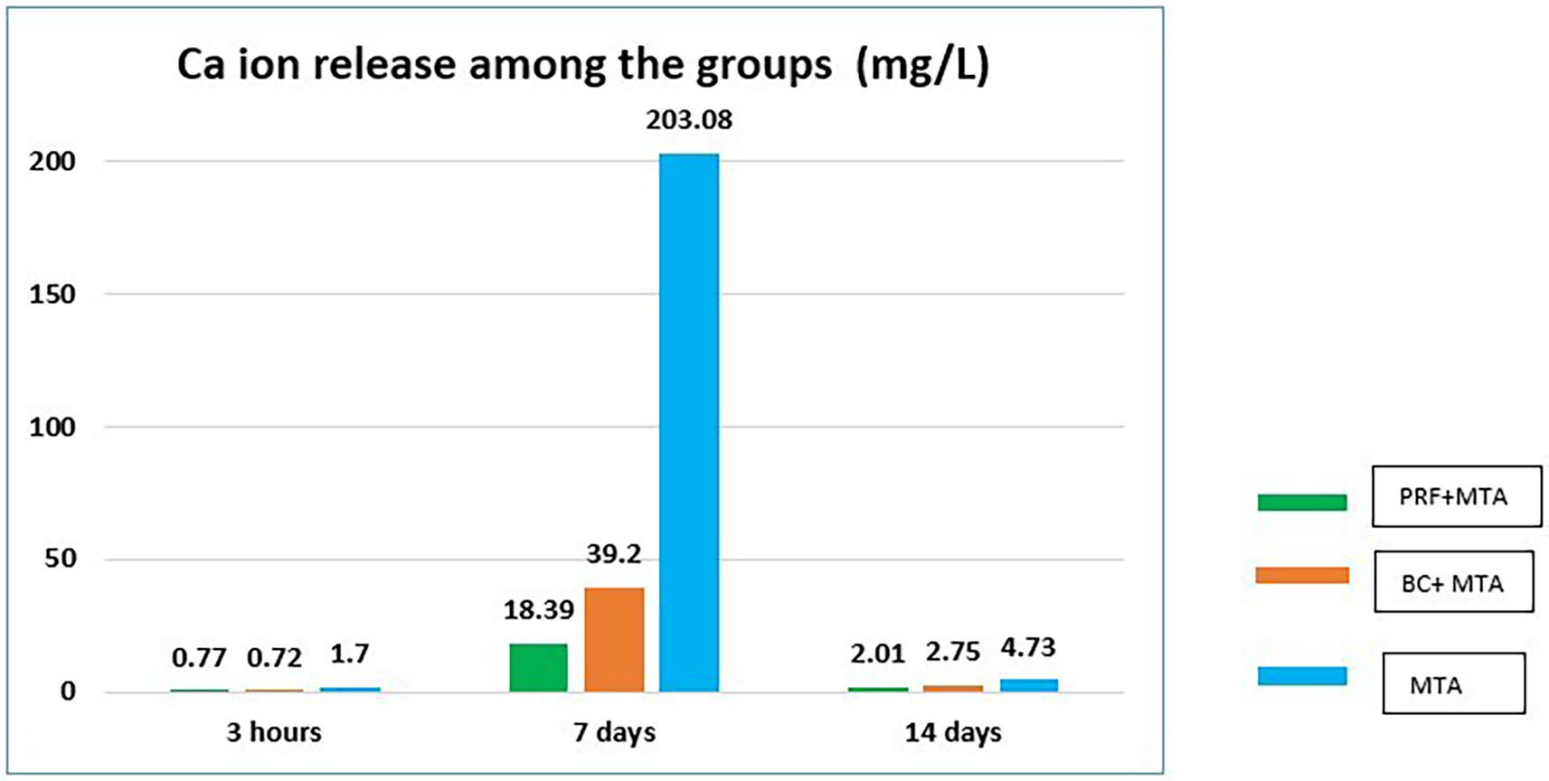
Bar graph representing Ca ^+^ ion release (mg/L) among the groups at different experimental periods.

## Discussion

The definitive objective of regenerative endodontic treatment modality for immature root apex with pulpal necrosis is continued root development. ^10^ Factors which influence the success of regeneration range from case selection, the technique followed, disinfection of the root canal, biomaterials, scaffolds and coronal seal. Immature teeth with open apex with a diameter of 1.1 mm or more respond favorably to REPs ^11^ as it permits the migration of mesenchymal stem cells into the canal space. The revascularization probability in such cases increases by approximately 18-34%. ^12^

Root conditioning with EDTA may neutralize the cytotoxicity provoked by NaOCl, enhancing cellular spreading and the liberation of bioactive molecules from the conditioned dentine. ^13^ It enabled the release of growth factors i.e. TGF-β present in dentin. TGF-β shows the ability to induce odontoblastic differentiation and contribute to dentinogenesis. ^14^

The blood collected from inside the root canal was found to have up to a 600-fold increase of CD73 and CD105 markers of mesenchymal stem cells compared to systemic blood. ^15^ Mechanically irritating the periapical tissues may cause discomfort to the patient and in some cases apical bleeding may not always be possible. Various scaffolds have been used as an alternative to (BC) which is rich in platelets, specifically PRP and PRF.

PRF contains physiological thrombin which creates symmetrical intersections in polymerized fibrin, which helps to release growth factors for up to 28 days. Additionally, their flexible fibrin network facilitates cell migration. Platelet derived growth factors (PDGF) and cytokines are abundant in platelets which play an important role in cellular differentiation. Thus, PRF scaffold has emerged as an effective biological tool in REPs. ^16^ Blood clot makes a weak fibrin mesh as compared to PRF. The use of biomaterials along with platelet concentrates containing fibrin and growth factors could lead to a paradigm shift in how endodontic regeneration can be achieved. MTA provides signalling molecules for the maturation of stem cells. ^17^ Ca ^+^ ions play a crucial role in the formation of mineralized hard tissues. Therefore, the study evaluated the release of Ca ^+^, a key element for regenerating pulp dentine complex.

### pH

An alkaline environment promotes osteogenic differentiation and bone formation. Mineralisation enzymes such as alkaline phosphatase peaks at pH 7.37 and significantly diminished under physiologic level. *In vitro* and *in vivo* research has shown that a pH above 8.0 inhibits the mineralization process. Therefore, various biological and molecular responses that influence repair and regeneration may depend on the local pH.

It is important to create an antibacterial environment at the tooth restoration interface or to control the residual bacteria in the canal space to reduce the risk of reinfection. ^18^

White MTA (WMTA) when in contact with tissue fluid, MTA dissolves and releases hydroxyl ions (OH ^-^) increasing the pH to 11-12 and contributing to reparative dentin formation. ^19^ Hydroxyl ions released from biomaterials also create a hostile environment for bacterial survival and proliferation reducing reinfection. Longer exposure to an alkaline environment could have induced potential degradation and a subsequent decrease in the detection of the growth factors. ^20^

All experimental groups showed an increase in pH over period of time. The highest alkaline pH was recorded in the control group, MTA with distilled water at seven days (11.54) and the lowest in PRF/MTA group (7.78). The difference in pH between PRF and blood clot at 3h and seven days (p < 0.001) was significant, but on the 14 ^th^ day these groups recorded similar values. Group 3 recorded a mean pH value of 10.76 at 3h, 11.54 on the seventh day and 11.14 on the 14 ^th^ day which was significantly different from the experimental groups. This is in accordance with the study. ^9^ In the same study, WMTA with blood recorded higher pH values in contrast to our study where the BC with MTA group recorded lower pH values. This could be because the experimental set up was different in that study: in that study, the MTA cylinder was prepared and immersed in blood whereas in our study, PRF/BC was placed over MTA and the pH recorded.

### Calcium ions

Calcium ions are known to act on osteoblasts and cementoblast cells, causing their differentiation and hard tissue mineralization. Hunter *et al*. (2018) reported that calcium ions released from biomaterials may influence the repair process, as they pass through the cell membranes by depolarization or activation of membrane-bound calcium channels. ^21^ Although Ca ^+^ ions are one of the major components released by MTA, the role of Ca ions in regenerative endodontics is largely underexplored. In the present study, the mean values for Ca ^+^ ion release from Group1 (PRF/MTA) and Group 2 (BC/MTA) was 0.77 mg L ^-1^ and 0.72 mg L ^-1^ respectively at 3h, which were not significantly different from each other. At seven days, Group 1 recorded a value of 18.39 mg L ^-1^ and Group 2 recorded a value of 39.20 mg L ^-1^. Though the mean Ca ^+^ ion release values of Group 2 were higher than for Group 1, it was not statistically significant at seven days (p ≤ 0.001). At day 14, there was significant reduction in the Ca ^+^ ion release where Group 1 recorded a value of 2.01 mg L ^-1^ and Group 2 recorded a value of 2.75 mg L ^-1^, which were again not significantly different from each other. Overall Group1 (PRF/MTA) showed the lowest release of calcium ions compared to Group 2 (BC/MTA) in14 days. At all experimental periods, Group 3 recorded the maximum Ca ^+^ ion release, which was statistically significant for both experimental groups. A previous study ^9^ evaluated Ca ^+^ ion release from WMTA with and without blood respectively, and reported that the group with WMTA and blood showed greater calcium ion release, which is contrary to what was recorded in our study. This could be because the experimental set up was different in that study, where a MTA cylinder was prepared and immersed in blood; in contrast, in our study, blood clot was placed over MTA and the ion release was recorded. To measure the release of Ca ^+^ ions, ICP-OES equipment was used, owing to its high sensitivity and stability for inorganic analysis. It provides rapid and multi-element analysis of the solutions. In this *in vitro* study, both experimental groups showed diffusion of Ca ^+^ ions from MTA through scaffolds at three hours, seven days and 14 days, though PRF/MTA allowed lower diffusion of Ca ^+^ ion as compared to the blood clot. However, various clinical studies have suggested that PRFs induce regeneration of pulp dentine complex better than blood clot as it is a rich source of platelets and growth factors. ^17^

There have been a limited number of studies evaluating the effect of PRF/BC with MTA on the pH and calcium ion release. In the present study, we observed that PRF and BC influenced the pH and Ca ^+^ ion release from MTA.

## Conclusions

While this study provides valuable insights into the interactions between MTA, PRF, and BC, further research, including in vivo studies and clinical trials, is needed. Additionally, it is crucial to evaluate various other interactions between PRF and different biomaterials. It can be concluded that PRF and BC adversely influenced the pH and Ca ^+^ ion release from the biomaterial MTA, and this effect decreased with time. Any interference with the release of calcium and hydroxy ions might impact the regenerative potential of MTA.

## Data Availability

Mendeley Data: pH and Calcium ion release from MTA when interacted with various substances, https://doi.org/10.17632/jr9sm49gj9.2. ^22^ This project contains the following underlying data:
‐ Mendley Dataset Values.docx ‐ Raw data.csv ‐ Results.csv Data are available under the terms of the Creative Commons Attribution 4.0 International license (CC-BY 4.0).
