# Supplementary material for: Comparative evaluation of pH and Ca + ion release from MTA on interaction with platelet-rich fibrin and blood clot: an in vitro study
Source: F1000Res. 2023 Oct 30;12:364. Originally published 2023 Apr 4. [Version 2] doi: 10.12688/f1000research.130227.2 (PMC10663652; doi:10.12688/f1000research.130227.2)
Supplement: Peer review history

# Reviewer report: Reviewer response for version 2

**DOI:** 10.5256/f1000research.158056.r219343

**Published:** 2023-11-21

**Authors:** Janet N Kirilova

**Affiliations:** 1 Medical University, Sofia, Bulgaria

Notes from the first version of the text have been corrected. I think this improves the quality of the article. I find it helpful and modern.

Is the work clearly and accurately presented and does it cite the current literature?

Partly

If applicable, is the statistical analysis and its interpretation appropriate?

Yes

Are all the source data underlying the results available to ensure full reproducibility?

Yes

Is the study design appropriate and is the work technically sound?

Partly

Are the conclusions drawn adequately supported by the results?

Yes

Are sufficient details of methods and analysis provided to allow replication by others?

Partly

Reviewer Expertise:

NA

I confirm that I have read this submission and believe that I have an appropriate level of expertise to confirm that it is of an acceptable scientific standard.

# Reviewer report: Reviewer response for version 2

**DOI:** 10.5256/f1000research.158056.r219342

**Published:** 2023-11-03

**Authors:** Amjad Abu Hasna

**Affiliations:** 1 São Paulo State University, São Paulo, Brazil

I was unable to locate the hypothesis.

 The other comments have been addressed, and I have no additional comments to provide.

Is the work clearly and accurately presented and does it cite the current literature?

Partly

If applicable, is the statistical analysis and its interpretation appropriate?

Yes

Are all the source data underlying the results available to ensure full reproducibility?

Yes

Is the study design appropriate and is the work technically sound?

Yes

Are the conclusions drawn adequately supported by the results?

Yes

Are sufficient details of methods and analysis provided to allow replication by others?

Yes

Reviewer Expertise:

Endodontics

I confirm that I have read this submission and believe that I have an appropriate level of expertise to confirm that it is of an acceptable scientific standard.

# Response

**Published:** 2023-11-05

**Authors:** Soumyadeep Ghosh

**Affiliations:** Manipal University, India

Thank you for your valuable comments Sir.

 1. Hypothesis is included at the last paragraph of introduction and it is stated as :-

 The regenerative technique should create an environment that favours disinfection and tissue regeneration. Inducing bleeding or use of PRF for facilitating root closure followed by placing a biomaterial may positively or negatively affect the pH and Ca ^+^ release. Knowing the release kinematics can improve our understanding of the regenerative process and help modify treatment strategies. This study thus explored the influence of PRF and BC on the pH and Ca ^+^ ion release from MTA.

# Reviewer report: Reviewer response for version 1

**DOI:** 10.5256/f1000research.142964.r199278

**Published:** 2023-09-07

**Authors:** Amjad Abu Hasna

**Affiliations:** 1 São Paulo State University, São Paulo, Brazil

I would like to extend my congratulations to the author for their dedication and hard work in conducting this study. Certainly, I will try to help the authors to improve their article in some comments

1. It is advisable to include the study's hypothesis towards the end of the introduction section for better contextualization.
2. Could you please provide the protocol number for the Human Research Ethics Committee of M S Ramaiah University of Applied Sciences in Bangalore, Karnataka?
3. A more concise conclusion is strongly recommended.
4. The authors have referenced a total of 19 sources, with merely 2 of them originating from the past five years. To enhance the scholarly foundation of this study, it is advisable to incorporate more up-to-date references into the bibliography. These recent sources can subsequently be leveraged to bolster the discussion, with a particular focus on highlighting the study's limitations.

Thank you for your attention to these matters.

Is the work clearly and accurately presented and does it cite the current literature?

Partly

If applicable, is the statistical analysis and its interpretation appropriate?

Yes

Are all the source data underlying the results available to ensure full reproducibility?

Yes

Is the study design appropriate and is the work technically sound?

Yes

Are the conclusions drawn adequately supported by the results?

Yes

Are sufficient details of methods and analysis provided to allow replication by others?

Yes

Reviewer Expertise:

Endodontics

I confirm that I have read this submission and believe that I have an appropriate level of expertise to confirm that it is of an acceptable scientific standard, however I have significant reservations, as outlined above.

# Response

**Published:** 2023-10-24

**Authors:** Soumyadeep Ghosh

**Affiliations:** Manipal University, India

Thank you for your valuable comments

 1. Hypothesis is included at the end of introduction and it is stated as :-

 The regenerative technique should create an environment that favours disinfection and tissue regeneration. Inducing bleeding or use of PRF for facilitating root closure followed by placing a biomaterial may positively or negatively affect the pH and Ca ^+^ release. Knowing the release kinematics can improve our understanding of the regenerative process and help modify treatment strategies. This study thus explored the influence of PRF and BC on the pH and Ca ^+^ ion release from MTA.

 2. Protocol Number for the Human Research Ethics Commitee of M S Ramaiah University of Applied Sciences, Bngalore, Karnataka is (EC-2018/PG/25). 

 3. Conclusion :- While this study provides valuable insights into the interactions between MTA, PRF, and BC, further research, including in vivo studies and clinical trials, is needed. Additionally, it is crucial to evaluate various other interactions between PRF and different biomaterials. It can be concluded that PRF and BC adversely influenced the pH and Ca ^+^ release from the biomaterial MTA, and this effect decreased with time. Any interference with the release of calcium and hydroxy ions might impact the regenerative potential of MTA.

 4. Recent references have been added :

Kucukkaya Eren S, Bahador Zırh E, Zeybek ND, Askerbeyli Örs S, Aksel H, Parashos P. Effect of benzalkonium chloride addition to EDTA on attachment and proliferation of dental pulp stem cells on dentin and on transforming growth factor-β1 release. Odontology. 2021 Apr;109:313-20.

dos Reis‐Prado AH, Abreu LG, Fagundes RR, Oliveira SD, Bottino MC, Ribeiro‐Sobrinho AP, Benetti F. Influence of ethylenediaminetetraacetic acid on regenerative endodontics: A systematic review. International Endodontic Journal. 2022 Jun;55(6):579-612.

Smoczer C, Yuth KR, Askar MA, Young LA, Paurazas SB. Growth Factors Released from Advanced Platelet-Rich Fibrin in the Presence of Calcium-Based Silicate Materials and Their Impact on the Viability and Migration of Stem Cells of Apical Papilla. Dentistry Journal. 2023 Sep 19;11(9):220

# Reviewer report: Reviewer response for version 1

**DOI:** 10.5256/f1000research.142964.r191859

**Published:** 2023-08-24

**Authors:** Janet N Kirilova

**Affiliations:** 1 Medical University, Sofia, Bulgaria

The proposed research is interesting. The results reflect changes in pH and emission of Ca ions alone with MTA administration or in combination with PRF and blood clots in the case of teeth with an open apex.

 To improve the work, I would recommend some changes such as:

**Introduction.** To formulate more precisely the purpose of the study.

**The methodology** does not specify the manufacturers of the different drugs. The methodology can be described in more detail for the individual groups.

 The PRF protocol differs from that of Shoukorun, whether the resulting product has the same properties – rich in cytokines and growth factors. The Shoukroun study is cited as number 8.

 The coronal end of the root was sealed with wax.  What kind and what is the purpose of sealing tightly or not?

**I** **n the discussion.** To discuss the importance of 17% EDTA for tertiary dentin formation.

 The sources of the last 5 years are relatively few.

 There is a need to improve English.

Is the work clearly and accurately presented and does it cite the current literature?

Partly

If applicable, is the statistical analysis and its interpretation appropriate?

Yes

Are all the source data underlying the results available to ensure full reproducibility?

Yes

Is the study design appropriate and is the work technically sound?

Partly

Are the conclusions drawn adequately supported by the results?

Yes

Are sufficient details of methods and analysis provided to allow replication by others?

Partly

Reviewer Expertise:

Conservative dentistry –glass-ionomer cements, regenerative endodontics-indirect and direct pulp cupping, digital dentistry-inlay, onlay, overlay

I confirm that I have read this submission and believe that I have an appropriate level of expertise to confirm that it is of an acceptable scientific standard, however I have significant reservations, as outlined above.

# Response

**Published:** 2023-10-24

**Authors:** Soumyadeep Ghosh

**Affiliations:** Manipal University, India

Thank you for your valuable contribution.

 1. Purpose of study -

 The regenerative technique should create an environment that favours disinfection and tissue regeneration. Inducing bleeding or use of PRF for facilitating root closure followed by placing a biomaterial may positively or negatively affect the pH and Ca ^+^ release. Knowing the release kinematics can improve our understanding of the regenerative process and help modify treatment strategies. This study thus explored the influence of PRF and BC on the pH and Ca ^+ ^release from MTA.

 2. The methodology - 

 Specification of manufacturers of different drugs -

 After mechanical instrumentation, the canals were irrigated with 1.5 % sodium hypochlorite (20mL, 5 min) (PERCAN N, SEPTODONT) ,5mL sterile physiological saline(0.9% NaCl) followed by a final rinse with 20 mL of 17% EDTA liquid (PRO Endo EDTA liquid, SS WHITE)  and dried using sterile paper points

 PRF protocol -

 Same as mentioned by Shoukron, 10 ml blood centrifuged immediately at 3000 revolutions/min (rpm) for 10 min, without anticoagulant. Resulting product has the same properties.

 Coronal end of the root was sealed with wax (Modelling wax , PYRAX ). The purpose of sealing it tightly was to form a matrix against which MTA could be condensedinto the prepared canal to obtain a thickness of 4mm. 

 3. Discussion 

 Importance of 17% EDTA-

 Root conditioning with 17% EDTA may neutralize the cytotoxicity provoked by NaOCl, enhancing cellular spreading and the liberation of bioactive molecules from the conditioned dentine. It enabled the release of growth factors i-e TGF- β present in dentin. TGF- β shows the ability to induce odontoblastic differentiation and contribute to dentinogenesis.

 Sources of last 5 year are incorporated.

 Shawky MO, Said Badr AE, El-Monem Tawfik MA, et al.: Evaluation of Calcium Ion Release from Two Calcium Silicate-Based Endodontic Materials. *SCIOL Biotechnol.* 2018;1:34–38.

 Taweewattanapaisan P, Jantarat J, Ounjai P, et al.: The effects of EDTA on blood clot in regenerative endodontic procedures. *J. Endod.* 2019 Mar 1;45(3):281–286. 30803535 10.1016/j.joen.2018.10.010

Kucukkaya Eren S, Bahador Zırh E, Zeybek ND, Askerbeyli Örs S, Aksel H, Parashos P. Effect of benzalkonium chloride addition to EDTA on attachment and proliferation of dental pulp stem cells on dentin and on transforming growth factor-β1 release. Odontology. 2021 Apr;109:313-20.

dos Reis‐Prado AH, Abreu LG, Fagundes RR, Oliveira SD, Bottino MC, Ribeiro‐Sobrinho AP, Benetti F. Influence of ethylenediaminetetraacetic acid on regenerative endodontics: A systematic review. International Endodontic Journal. 2022 Jun;55(6):579-612.

Smoczer C, Yuth KR, Askar MA, Young LA, Paurazas SB. Growth Factors Released from Advanced Platelet-Rich Fibrin in the Presence of Calcium-Based Silicate Materials and Their Impact on the Viability and Migration of Stem Cells of Apical Papilla. Dentistry Journal. 2023 Sep 19;11(9):220

 Improvement in English - Rectified.
